# Systems analysis of latent HIV reversal reveals altered stress kinase signaling and increased cell death in infected T cells

**DOI:** 10.1038/s41598-017-15532-0

**Published:** 2017-11-23

**Authors:** Linda E. Fong, Endah S. Sulistijo, Kathryn Miller-Jensen

**Affiliations:** 1 Department of Biomedical Engineering, Yale University, New Haven, CT USA; 2 Department of Molecular, Cellular, and Developmental Biology, Yale University, New Haven, CT USA

## Abstract

Viral latency remains the most significant obstacle to HIV eradication. Clinical strategies aim to purge the latent CD4+ T cell reservoir by activating viral expression to induce death, but are undercut by the inability to target latently infected cells. Here we explored the acute signaling response of latent HIV-infected CD4+ T cells to identify dynamic phosphorylation signatures that could be targeted for therapy. Stimulation with CD3/CD28, PMA/ionomycin, or latency reversing agents prostratin and SAHA, yielded increased phosphorylation of IκBα, ERK, p38, and JNK in HIV-infected cells across two *in vitro* latency models. Both latent infection and viral protein expression contributed to changes in perturbation-induced signaling. Data-driven statistical models calculated from the phosphorylation signatures successfully classified infected and uninfected cells and further identified signals that were functionally important for regulating cell death. Specifically, the stress kinase pathways p38 and JNK were modified in latently infected cells, and activation of p38 and JNK signaling by anisomycin resulted in increased cell death independent of HIV reactivation. Our findings suggest that altered phosphorylation signatures in infected T cells provide a novel strategy to more selectively target the latent reservoir to enhance eradication efforts.

## Introduction

Cellular reservoirs infected with latent human immunodeficiency virus-1 (HIV) are the primary obstacle to HIV eradication^1,2^. The most promising therapeutic approach is to purge the latent HIV reservoir residing in CD4+ T cells with latency reversing agents (LRAs)—proteins or small molecules that promote activation of the latent virus^3^. A major limitation of this approach is that LRAs cannot be targeted to latently infected cells, and efforts to identify biomarkers that distinguish latently infected T cells from uninfected cells have had mixed success^4–6^.

One reason biomarkers of latent HIV infection are difficult to identify is that biological changes which cause disease often do not produce clear differences in protein levels that can be observed in a basal state, but instead affect interactions between proteins^7^. For this reason, stimulating diseased cells and following the dynamics of protein activation over time has proved to be a successful way to differentiate between healthy and diseased cells in cancer^8^ and type 1 diabetes^9^ and to therapeutically target the disease state^10^. There is evidence that latent HIV-infected T cells exhibit virus-induced changes, including chromatin-mediated transcriptional silencing and altered activities of select kinases^5,11,12^, which might affect signaling in latently infected cells following stimulation in a manner similar to a disease state. This raises the possibility–as yet untested–that T cell signaling networks are altered by latent HIV infection or by viral protein expression upon latency reversal, and that these differences could be targeted for HIV eradication.

In this study, we used a systems biology approach to explore if latent HIV-infected T cells display altered signaling upon acute stimulation of T cell activation. T cell activation via T cell receptor (TCR) stimulation or treatment with phorbol 12-myristate 13-acetate/ionomycin (PMA/I) strongly activates HIV gene expression through the phosphorylation of multiple signaling pathways. These pathways include the extracellular regulated kinase (ERK) pathway, the nuclear factor-κB (NF-κB) pathway, and the phosphatidylinositol 3-kinase (PI3K)/AKT pathway, which activate downstream transcription factors that induce HIV gene expression^13–17^. While broad T cell activation is not a viable strategy in patients^18,19^, LRAs such as prostratin and bryostatin-1 target similar pathways but can induce viral expression without global T cell activation^14,20–24^.

We measured time-dependent phosphorylation signatures in uninfected and infected T cells following stimulation with CD3/CD28, PMA/I and prostratin ± SAHA. We observed increased phosphorylation across multiple pathways in infected cells as compared to uninfected cells for both primary CD4+ cultured central memory T cells and Jurkat T cell models. Some signaling differences were present in infected cells maintaining latent virus, while others were coincident with viral protein expression. Computational data-driven analysis demonstrated that systems-level changes in phosphorylation signatures following stimulation were sufficient to differentiate infected cells from uninfected cells. Regression models, together with experimental validation, revealed that latently infected cells were sensitized to pro-death signaling via the p38 and JNK MAPK pathways and that the expression of viral proteins increased this effect. We propose that targeting modified systems-level signaling in latently infected cells provides a clinically promising strategy to improve LRA specificity and efficacy.

## Results

### Kinase phosphorylation signatures following T cell activation are different between latent HIV-infected and uninfected primary T_CM_ cells *in vitro*

**Figure 1:**
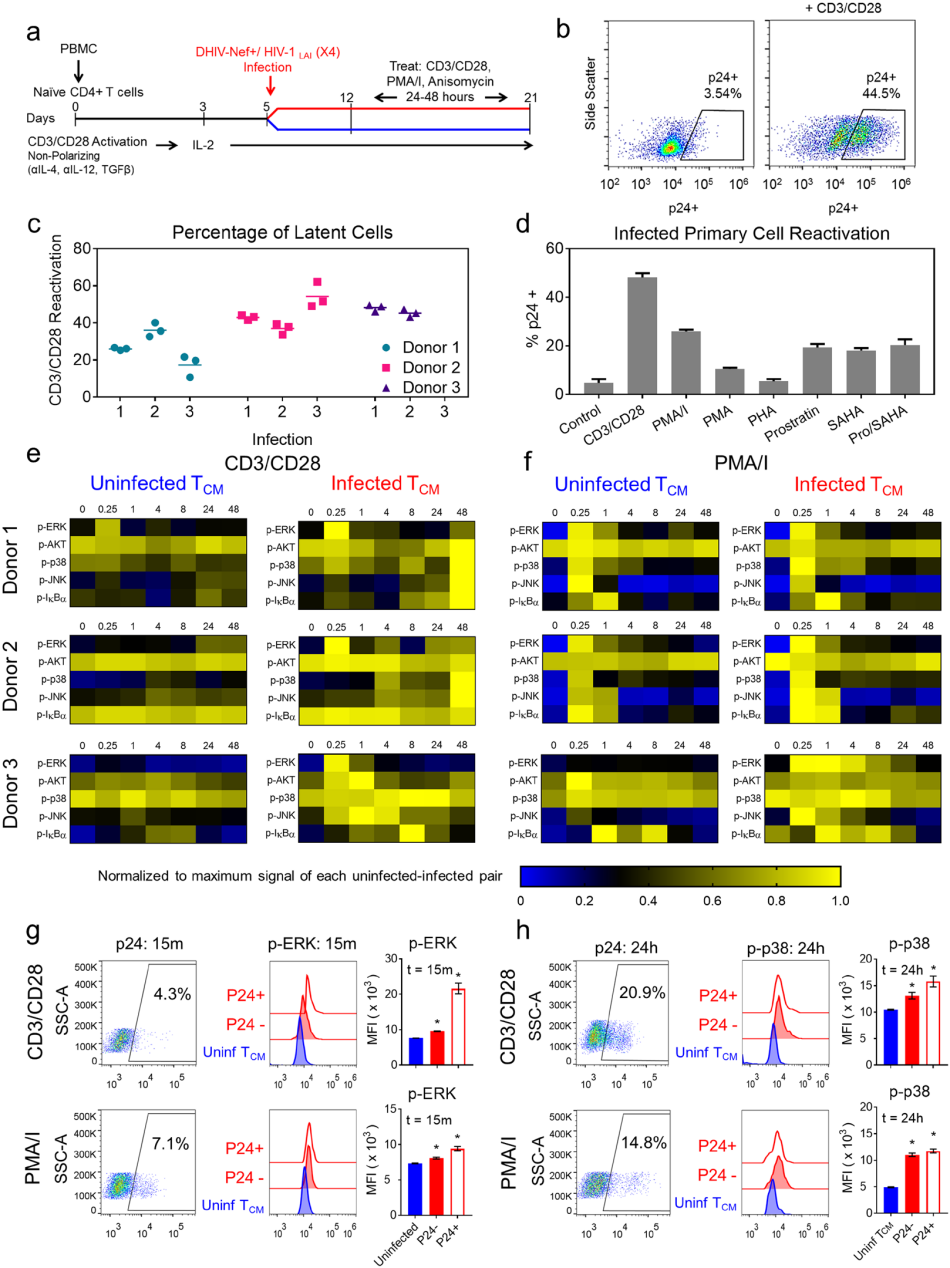
Latently infected CD4+ T_CM_ cells exhibit altered phosphorylation signatures relative to uninfected cells following T cell activation. (**a**) Experimental timeline of *in vitro* primary CD4+ T_CM_ cell model generation. After DHIV-Nef +/ HIV-1 _LAI_ (X4) infection on day 5, uninfected and infected cells are cultured in parallel. (**b**) Scatter plots of Gag p24 expression in CD4+ T_CM_ cells before (left) and after stimulation with CD3/CD28 (right) measured by flow cytometry. Representative data from donor 2; n = 10,000 cells. (**c**) Range of latent infection levels for replicate infections across 3 donors as estimated by CD3/CD28 stimulation of viral reactivation. (**d**) Viral reactivation after 48 hours of stimulation across a panel of LRAs. Data presented as means ± SD (n = 3) for donor 2. (**e**,**f**) Heatmaps depict phospho-protein time courses following CD3/CD28 (**e**) or PMA/I stimulation (**f**) in uninfected and infected CD4+ T_CM_ cells for 3 donors over 48 hours measured by bead-based immunoassay. Phospho-signals were measured in biological triplicate and 90% of measurements had an SD < 18%. Data are normalized to the maximum signal for each paired stimulation time course (uninfected and infected cells). Raw data are provided in Supplementary Datasets S1–S6. (**g**,**h**) Flow cytometry analysis of p-ERK at 15 minutes (**g**) and p-p38 at 24 hours (**h**) in uninfected, latently infected (p24−) and infected/reactivaing (p24+) T_CM_ cells following CD3/CD28 (top) or PMA/I stimulation (bottom). Gating for p24 (left plots) and histogram intensities for each population (middle plots) are shown for a representative donor. Mean fluorescence intensity (MFI) of phospho-protein levels (right plots) presented as means ± SD for 3 donors.

To evaluate if latent HIV infection alters signal transduction in infected T cells, we used an *in vitro* human primary CD4+ T cell model (Fig. 1a). Cultured cells were differentiated by TCR stimulation under non-polarizing conditions to induce a central memory T cell (T_CM_) phenotype and subdivided for infection on day 5^25,26^. Cells were infected with a CXCR4-pseudotyped, non-replication competent HIV construct (DHIV-Nef +/ HIV-1 _LAI_ (X4)) that encodes for all viral proteins but carries an inactivating deletion in Env^25,26^. Uninfected and infected T_CM_ cell populations were then cultured in parallel for at least one week post-infection, allowing both populations to return to a quiescent state. In the quiescent state, basal virus expression in the infected T_CM_ cell population is predominantly latent (<5% of cells express virus). Stimulation with CD3/CD28 for 48 hours induced cellular activation similarly in both populations (Supplementary Fig. S1) and resulted in significant intracellular expression of Gag p24 in infected cells (Fig. 1b).

The T_CM_ HIV latency model produces a high ratio of latently infected cells relative to uninfected cells as compared to other primary latency models^26^, which increases the likelihood of measuring signaling differences between uninfected and latently infected cells. The size of the latently infected T_CM_ cell population was estimated by the fraction of cells that expressed HIV in response to CD3/CD28 stimulation and ranged from approximately 20% to 50%, depending on the viral infection and donor (Fig. 1c). The remaining 50% to 80% of T_CM_ cells were either uninfected or contain latent virus that does not reactivate. The efficiency of latent viral reactivation varied for different LRAs as previously described^25,27^ (Fig. 1d). Overall, the primary T_CM_ latency model enables a direct comparison between populations of pure uninfected cells and latently infected cells in a background of uninfected cells, where the only affected variable is viral infection.

We collected a multivariate set of signaling measurements to examine whether latent infection perturbs the host-cell signaling response. We focused on T cell receptor (TCR) stimulation by CD3/CD28 and stimulation by PMA/I, which elicit strong viral reactivation (Fig. 1d). We monitored activation of five protein kinases that are perturbed during viral infection and reactivation, including phospho-ERK (p-ERK), p-AKT, p-p38, p-JNK, and p-IκBα (Supplementary Table S1). Levels of phosphorylation were measured at 0.25, 1, 4, 8, 24, and 48 hours following activation with CD3/CD28 or PMA/I in T_CM_ cells isolated from three donors infected *ex vivo* (Fig. 1c and Supplementary Table S2).

There were no significant differences in basal phosphorylation between uninfected and infected T_CM_ cells prior to stimulation (Supplementary Fig. S2a), but we observed significant differences in phospho-protein levels following TCR stimulation with CD3/CD28 (Fig. 1e). Phosphorylation was consistently higher in infected T_CM_ cells as compared to uninfected T_CM_ cells following TCR stimulation for all phospho-proteins. In infected T_CM_ cells, we observed increased levels of p-ERK shortly after stimulation and elevated levels of p-p38, p-JNK, and p-IκBα at later time points across multiple donors.

Inducing T cell activation chemically with PMA/I (i.e., without TCR engagement) also revealed differences in phosphorylation signatures between uninfected and infected T_CM_ cells (Fig. 1f). Similar to CD3/CD28-induced signaling, infected T_CM_ cells stimulated with PMA/I exhibited higher peak levels of p-ERK and elevated levels p-p38. We observed donor-dependent differences in p-AKT, p-JNK, and p-IκBα following PMA/I stimulation.

The observed changes in signaling could be caused by the establishment and maintenance of latent HIV infection prior to reactivation, or by interactions with viral proteins expressed upon latency reversal. To begin to distinguish between these possibilities, we focused on early p-ERK signaling and late p-p38 signaling, which were both elevated in infected T_CM_ cell populations (Fig. 1e,f). By analyzing the levels of these proteins simultaneously with Gag p24 expression by flow cytometry, we could separate the contribution of virus-expressing cells from cells maintaining latent virus. Following stimulation with CD3/CD28 or PMA/I, p-ERK levels at 15 minutes were slightly higher in latently infected cells (p24−) and much higher in virus-expressing cells (p24+ ) relative to uninfected cells (Fig. 1g). The significant increase in p-ERK levels in p24+ cells suggested that viral proteins contribute substantially to increased ERK signaling, even though the p24+ fraction is very low (Fig. 1g). In contrast, p-p38 was similarly elevated in both latent (p24−) and virus-expressing (p24+) cells at 24 hours following both stimulations (Fig. 1h), demonstrating that stress kinase signaling is altered even when the virus is latent.

### Kinase phosphorylation signatures following T cell activation are different between uninfected and infected cells in a Jurkat model of latency

**Figure 2:**
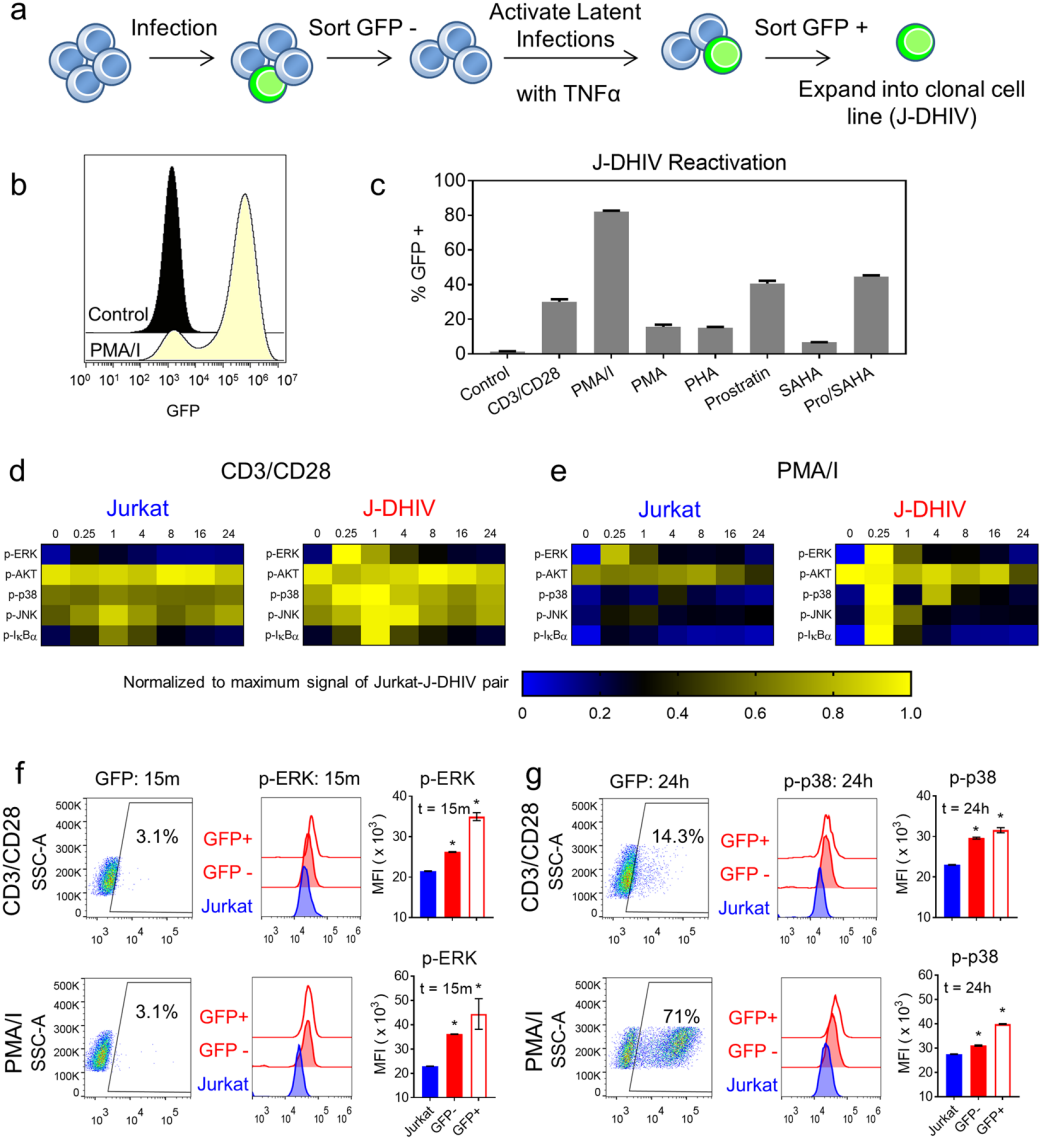
Altered phosphorylation signatures observed in a latently infected Jurkat cell line model following T cell activation. (**a**) Schematic depicts the sorting strategy for generating a clonal DHIV-GFP/ HIV-1 _LAI_ (X4)-infected Jurkat cell line (J-DHIV). (**b**) Histogram of GFP expression in the J-DHIV cells before (black) and after stimulation with PMA/I (yellow) measured by flow cytometry. (**c**) Viral reactivation levels after 24 hours of stimulation across a panel of LRAs. Data presented as means ± SD for 3 biological replicates. (**d**,**e**) Heatmaps depict phospho-protein time courses following CD3/CD28 or PMA/I stimulation in Jurkat and J-DHIV cells over 24 hours measured by bead-based immunoassay. Phospho-signals were measured in biological triplicate and 90% of measurements had an SD < 14%. Data are normalized to the maximum signal for each paired stimulation time course (uninfected and infected cells). Raw data are provided in Supplementary Datasets S7–S8. (**f**–**g**) Flow cytometry analysis of p-ERK at 15 minutes (**f**) and p-p38 at 24 hours (**g**) in uninfected Jurkat, latently infected (GFP-) and infected/reactivating (GFP+) J-DHIV cells following CD3/CD28 (top) or PMA/I stimulation (bottom). Gating for p24 (left plots) and histogram intensities for each population (middle plots) are shown. MFI of phospho-protein levels (right plots) presented as means ± SD for 3 biological replicates.

Due to the high degree of variability that experimental HIV latency models exhibit in response to LRA stimulation^27^, we compared our results to a Jurkat T cell line model of latency and its uninfected parental Jurkat cell line to identify conserved changes. The latent cell line was infected with DHIV-GFP/ HIV-1 _LAI_ (X4), the same virus used to infect primary cultured T_CM_ cells but with GFP in place of Nef^28^. The GFP expression marker allowed us to isolate our cell line model by the same strategy used to establish the widely-studied J-Lat latency models^29^. To minimize differences introduced by cell culture history, we sorted and expanded the uninfected and infected cell lines in parallel (referred to as Jurkat and J-DHIV, respectively; Fig. 2a).

Basal viral GFP expression was very low for J-DHIV but more than 80% of cells expressed GFP following PMA/I stimulation (Fig. 2b). Responses to other LRAs were similar to the previously established J-Lat lines^27^, with the exception of CD3/CD28 (Fig. 2c). J-DHIV cells responded robustly to CD3/CD28 stimulation, with 20–25% of cells expressing GFP, allowing us to directly compare signaling activation in response to both CD3/CD28 and PMA/I stimulation between the two latency models.

We measured CD3/CD28 and PMA/I-stimulated signaling in Jurkat and J-DHIV cells over 24 hours due to the more rapid viral activation exhibited in cell line models. Consistent with primary cultured T_CM_ cells, Jurkat and J-DHIV cells showed similar basal phosphorylation levels (Supplementary Fig. S2b). Stimulation with CD3/CD28 resulted in increased p-ERK, p-p38, p-JNK, and p-IκBα in J-DHIV cells relative to Jurkat cells (Fig. 2d). The general increase in phospho-protein levels observed in J-DHIV cells was similar to our observations in the primary infected T_CM_ model, although the dynamics were somewhat different. Treatment with PMA/I clearly showed elevated phospho-signaling for all 5 proteins in J-DHIV cells (Fig. 2e).

We again analyzed early p-ERK signaling and late p-p38 signaling simultaneously with Gag p24 levels by flow cytometry to assess the contribution of viral protein expression to changes in signaling (Fig. 2f,g). Similar to our observations in primary T_CM_ cells, p-ERK levels were increased in both latent (GFP-) cells and virus-expressing (GFP+ ) cells relative to Jurkat cells, with p-ERK levels much higher in virus-expressing cells. Latent and virus-expressing cells showed similar increased levels of p-p38 following CD3/CD28 treatment, confirming that latent virus contributed to elevated stress kinase signaling even in the absence of viral protein expression. Overall, our results demonstrate that latent HIV infection and the subsequent reactivation of viral proteins increase phospho-signaling following T cell activation, as observed across two experimental models of latency.

### Kinase phosphorylation signatures following T cell activation discriminate between infected and uninfected cells

Viral infection can be viewed as a systems-level perturbation^30^, and thus we sought to analyze network-level differences between the phosphorylation patterns of infected and uninfected cells. Although infected cells generally exhibited increased phosphorylation following stimulation, we observed significant variability in the level and timing of phosphorylation across experimental models and pathways. We therefore wanted to assess whether dynamic phosphorylation signatures would be sufficient to separate infected cells from uninfected cells within each treatment. Successful classification of infected cells based on dynamic phosphorylation signatures would suggest a conserved systems-level change in signaling despite variability across donors and experimental latency models.

**Figure 3:**
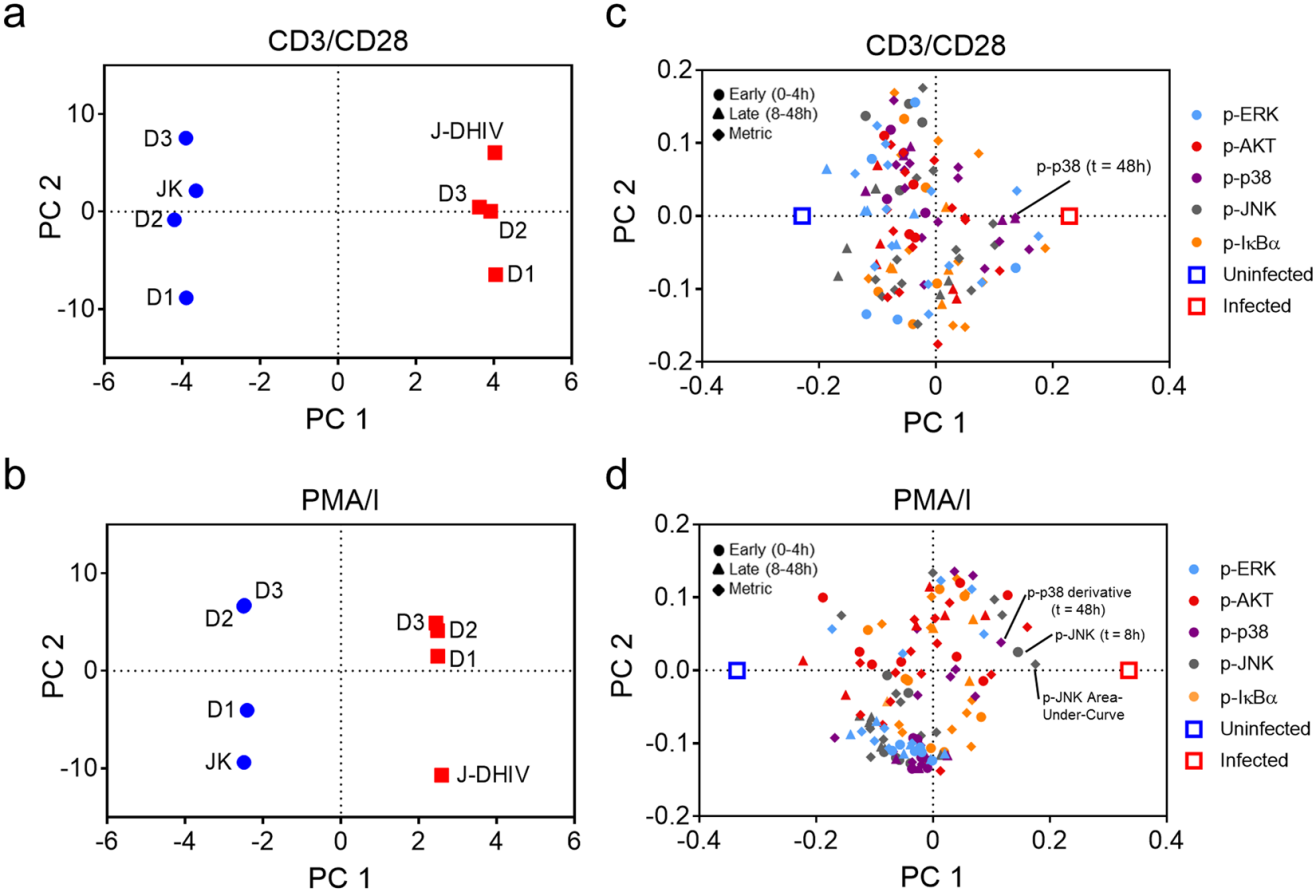
PLS-DA model of dynamic phosphorylation signatures classifies uninfected and infected T cells. PLS-DA models comprising three principal components were calculated from phospho-protein time courses for uninfected and latently infected T cells treated with CD3/CD28 (R^2^X = 0.95, R^2^Y = 0.99, Q^2^ = 0.61) or PMA/I (R^2^X = 0.93, R^2^Y = 0.99, Q^2^ = 0.80) (**a,b**) Model scores projected onto PC1 versus PC2 for uninfected (blue) and infected (red) cells for both primary T_CM_ cells from three donors (D1, D2, D3), and Jurkat (JK)/J-DHIV cell lines treated with CD3/CD28 (**a**) or PMA/I (**b**). (**c**,**d**) Model loadings of phospho-proteins and host-cell classification (uninfected, blue square; infected, red square) projected onto PC1 versus PC2 for CD3/CD28 treatment (**c**) or PMA/I treatment (**d**). Early time points (0–4 hours), circles; late time points (8–48 hours), triangles; time-dependent metrics, filled squares. Color codes of signaling pathways labeled on graph.

Projection-based regression methods are useful to condense complex, time-dependent data in order to reveal biological meaning^30–32^. Here we applied partial least squares-discriminant analysis (PLS-DA) to determine if differences in multivariate phosphorylation signals are sufficient to classify infection status (Fig. 3). This supervised classification algorithm identifies linear combinations of the measured signals that maximally separate pre-defined groups within a biological dataset (in this case, infected versus uninfected cells) and then projects these onto a new set of “super” variables called principal components. In addition to the signaling activation data at each time point, our PLS-DA models incorporated several data-derived signaling metrics to capture time-dependent features encoded in the signaling time courses^31,33^. We reasoned that such features might be important because phenotypic changes in cell behavior can arise from integrated signal processing, as well as from the relative level of signal activation^34–36^.

We tested if a PLS-DA model could distinguish between uninfected and infected cells for both primary cultured T_CM_ and cell line experimental models in response to CD3/CD28 or PMA/I stimulation. For CD3/CD28 treated cells, the optimal three-principal-component-model captured a majority of the variation in the dataset (R^2^Y = 0.99) and showed high predictive ability such that each individual cell-treatment combination could be robustly classified based on a PLS-DA model built from the remaining treatment datasets (Q^2^ = 0.61) (Fig. 3a). The PLS-DA model was similarly able to classify PMA/I treated cells (R^2^Y = 0.99, Q^2^ = 0.80) (Fig. 3b). Thus, PLS-DA showed that the phosphorylation signatures following T cell activation were sufficient to distinguish infected and uninfected cells across both primary cultured T_CM_ cells and Jurkat T cell line models of latency.

We visualized the relationship between signaling variables and infection status by plotting their respective model weights, or the contribution of each signaling variable to the principal components of the model (Fig. 3c–d). Principal component 1 (PC1) loadings indicated the signals most important for discriminating between infected and uninfected cells. Examination of PC1 loadings across both the CD3/CD28 and PMA/I models indicated that no individual pathway systematically distinguished between uninfected and latently infected cells, but some trends were observed. Metrics related to p-JNK and p-p38 at late time points (8–48 hours) after stimulation were more strongly associated with infected cells (positive on PC1), while p-AKT signaling variables were more strongly associated with uninfected cells (negative on PC1). Overall, we conclude that uninfected and latently infected cells exhibit distinct network-level phosphorylation patterns following T cell activation.

### Phospho-signaling differences in infected cells are observed after stimulation with prostratin +/− SAHA

We next investigated whether network-level changes in phospho-signaling were present in infected cells stimulated without global T cell activation. To test this, we measured activation of the same five signaling proteins following stimulation with the PKC agonist prostratin and the histone deacetylase (HDAC) inhibitor SAHA, alone and in combination. In primary cultured T_CM_ cells, prostratin stimulation increased levels of all measured phospho-signals in latently infected cells (Supplementary Fig. S3a). In the J-DHIV cell line, treatment with prostratin yielded increased signaling for all phospho-proteins except p-JNK (Supplementary Fig. S3b). The administration of prostratin in combination with SAHA (prostratin/SAHA) did not significantly change phosphorylation patterns compared to prostratin alone (Supplementary Fig. S3a,b).

A PLS-DA model built from prostratin and prostratin/SAHA conditions reliably discriminated between uninfected and infected primary T_CM_ cells and cell lines (Supplementary Fig. S3c,d; R^2^Y = 0.99, Q^2^ = 0.82). Visualization of the model weights showed that p-p38 and p-JNK variables associated more closely with infected cells, while p-ERK and p-IκBα variables associated with uninfected cells. Altogether, this multivariate analysis further supports the existence of global changes in host T cell signaling networks, induced by the establishment or reactivation of latent HIV, that may impact responses to LRA treatment.

### PLSR modeling of multivariate signaling data links stress kinase signaling pathways to increased cell death following T cell activation in infected cells

We sought to explore the possibility of targeting dysregulated signals to specifically increase cell death in latently infected cells. This is an important clinical problem because eradicating the latent T cell reservoir relies on the premise that cells harboring reactivated virus will die from viral cytopathic effects or host immune targeting following viral induction. However, there is evidence that viral cytopathic effects are not sufficient to induce cell death following reactivation of infected resting CD4+ T cells, even in the presence of autologous cytolytic T lymphocytes (CTLs) from patients on ART^37^.

**Figure 4:**
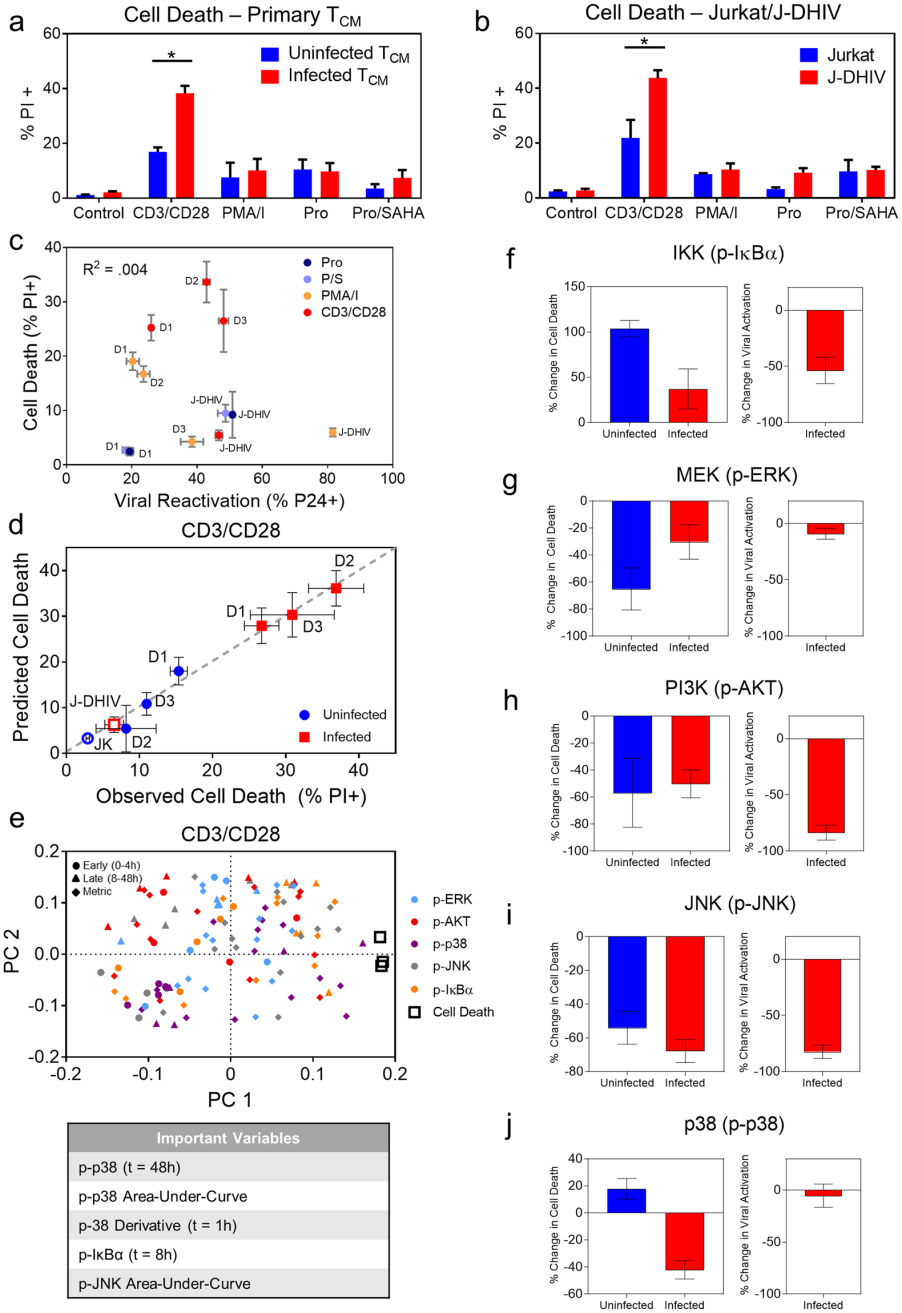
Inhibition of p38 selectively modulates cell death in infected cells following stimulation with CD3/CD28. (**a**,**b**) Summary of cell death across treatments for uninfected (blue) and infected (red) CD4+ T_CM_ cells (**a**) and Jurkat/J-DHIV cell lines (**b**). Data presented as means ± SD for 3 biological replicates. (**c**) Correlation plot of cell death (PI+) and viral reactivation (p24+) for the panel of LRAs presented in (**a**,**b**) measured simultaneously by flow cytometry. Data presented as means ± SD for 3 biological replicates. (**d**,**e**) Partial least squares regression (PLSR) model was calculated from phospho-protein time courses and cell death outcomes for uninfected and infected T cells treated with CD3/CD28. (**d**) Predicted versus observed cell death levels measured across primary T cells and cell lines (R^2^X = 0.55, R^2^Y = 0.99, Q^2^ = 0.68). Horizontal error bars indicate mean ± SD of 3 biological replicates. Vertical error bars indicate model uncertainty calculated by jack-knifing. (**e**) Model loadings of phospho-proteins and cell death (open black squares) projected onto PC1 versus PC2. Early time points (0–4 hours), circles; late time points (8–48 hours), triangles; time-dependent metrics, filled squares. Color codes of signaling pathways labeled on graph. Table lists the top 5 VIP values positively associated with cell death. (**f**–**j**) Cell death (left plots) for primary uninfected T_CM_ cells (blue) and infected T_CM_ cells (red) and viral reactivation in infected T_CM_ cells (right plots) in response to stimulation with CD3/CD28 and ACHP (**f**), PD 98059 (**g**), LY 294002 (**h**), SP 600125 (**i**), and SB 203580 (**j**). Percentage of PI+ cells and p24 + cells was measured by flow cytometry and the absolute change in percent cell death or viral reactivation was calculated relative to the uninhibited control. Data are presented as means ± SD for 5 biological replicates across 2 donors. *p < 0.05 by Student’s t test.

We analyzed cell death in response to the panel of LRA stimulations for which we observed network-level changes in phosphorylation signatures in infected T cells (Fig. 3). Only CD3/CD28 stimulation produced a significant difference in cell death between uninfected and infected cells for both primary cultured T_CM_ and Jurkat/J-DHIV cell line models as measured by propidium iodide (PI) staining (Fig. 4a,b). Cell death predominantly occurred via apoptosis because PI measurements correlated closely to cleaved caspase 3 immunostaining (Supplementary Fig. S4a). We did not observe any correlation between reactivation of latent virus and cell death in infected cells (Fig. 4c), suggesting that the difference in cell death observed for CD3/CD28 was not simply due to increased viral reactivation. Therefore, we focused on exploring the relationship between network-level differences in phospho-signaling caused by perturbing latent HIV infection and T-cell activation-induced cell death after CD3/CD28 stimulation.

To identify the most promising signaling targets, we built a partial least squares regression (PLSR) model for CD3/CD28 stimulation that correlated the phospho-signaling dynamics in uninfected and infected cells (Fig. 1e and Fig. 2d) to the cell death responses that were measured in parallel (Fig. 4a,b). PLSR is a statistical regression technique similar to PLS-DA, where the independent X-variables are linearly optimized to maximally explain the variance of the response Y-variables^38^. These variables are then projected onto a new principal component space. Such models can be used to correlate the effects that independent variables (e.g., phospho-signals) have on dependent variables (e.g., the percentage of dying cells), and thus can serve as a hypothesis generating “causal” modeling tool.

**Figure 5:**
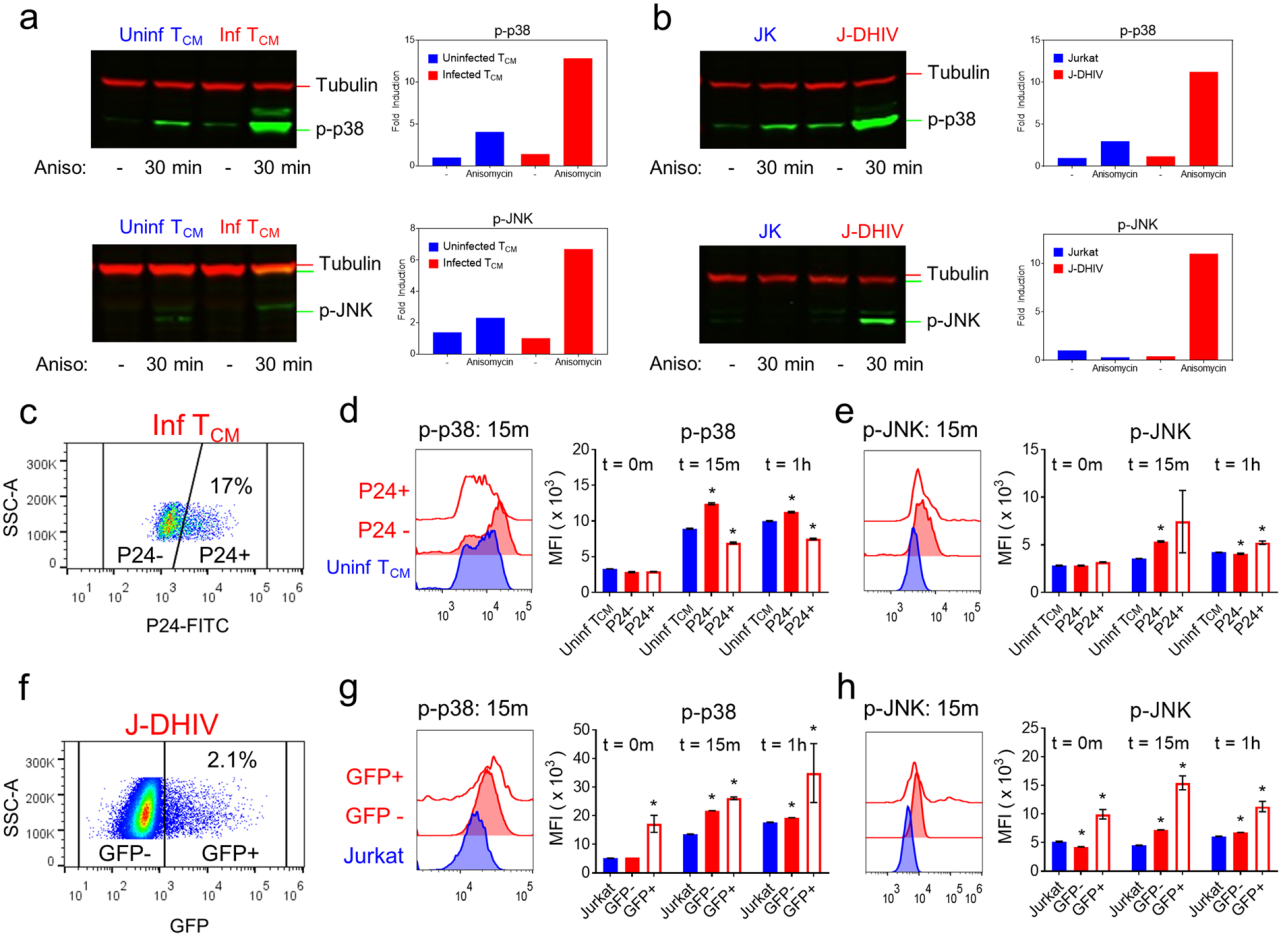
Latently infected cells exhibit increased p38 and JNK stress kinase signaling in response to anisomycin. (**a**,**b**) Western blots for p-p38 (top) and p-JNK (bottom) expression^46^ with and without anisomycin treatment (30 minutes) in uninfected (left) and infected (right) primary T_CM_ cells (**a**), and Jurkat (JK, left) /J-DHIV cells (right) (**b**). Mean intensities were normalized to the alpha-Tubulin loading control^47^ and fold increase in mean intensity was quantified (right plot). Cropped images shown here, full-length blots are presented in Supplementary Fig. S6. (**c–h**) Flow cytometry analysis of virus expression (**c**), p-p38 (**d**) and p-JNK (**e**) levels. (**c**) Gating for p24 and (**d,e**) histogram intensities (left plot) is shown for primary T_CM_ cells. MFI of phospho-protein levels at 0 m, 15 m, and 1 hour following stimulation with anisomycin for uninfected (blue), latent p24− (red-filled) and p24+ (red-open) primary T_CM_ cells is presented as means ± SD for 3 donors. (**f**) Gating for GFP and (**g**,**h**) histogram intensities (left plot) is shown for Jurkat/J-DHIV cells. MFI of phospho-protein levels at 0 m, 15 m, and 1 hour following stimulation with anisomycin for uninfected Jurkat (blue), latent GFP- (red-filled) and GFP+ (red-open) J-DHIV cells is presented as means ± SD for 3 biological replicates.

When we compared CD3/CD28-induced cell death predicted by the PLSR model to experimental measurements, the model showed robust predictive capability (Fig. 4d; R^2^Y = 0.96, Q^2^ = 0.68). Inspection of the VIPs identified p-p38 and p-JNK metrics among the top five variables positively associated with cell death following CD3/CD28 stimulation (Fig. 4e). Based on this observation, we hypothesized that increased p38 and JNK activity may be causally linked to increased T cell activation-induced cell death observed in infected cells.

To test the role of each pathway in modulating cell death, we applied kinase signaling inhibitors and then measured changes in cell death and viral reactivation following 48 hours of CD3/CD28 stimulation. Inhibition of the IKK/IκBα pathway significantly increased cell death in both uninfected and infected T_CM_ cells, even though viral reactivation was reduced (Fig. 4f). Inhibition of MEK/ERK and PI3K/AKT pathways resulted in similar reductions in cell death (~50%) in both uninfected and infected T_CM_ cells, despite having very different effects on viral reactivation (Fig. 4g,h). PI3K/AKT inhibition decreased viral reactivation by more than 80%, while MEK/ERK inhibition had little effect. Inhibition of JNK reduced cell death slightly more in infected primary T_CM_ cells than uninfected, though viral reactivation was reduced to similar levels as for PI3K/AKT (Fig. 4i). Interestingly, p38 inhibition differentially affected cell death, causing a small increase in cell death for uninfected T_CM_ cells, but decreasing death by ~40% in infected T_CM_ cells (Fig. 4j). The observed decrease in cell death was not coupled to a decrease in viral reactivation. We found that p38 inhibition also reduced cell death more in J-DHIV cells than in Jurkat cells (Supplementary Fig. S5a,b). Thus we conclude that p38 differentially regulates cell death following CD3/CD28 stimulation in infected versus uninfected cells.

### Latently infected cells are sensitized to the pro-death signaling of p38 and JNK

Based on the strong correlation of p-p38 and p-JNK metrics with cell death in the PLSR model and the differential cell death response to p-p38 inhibition, we hypothesized that infected cells may be sensitized to targeted activation of these pathways. To test this, we used the antibiotic anisomycin, a potent activator of the p38 and JNK pathways^39^. We measured the phosphorylation of p38 and JNK following treatment with anisomycin for 30 minutes, and found that infected cells exhibited increased phosphorylation of both kinases in primary T_CM_ cells and in J-DHIV cells (Fig. 5a,b, Supplementary Fig. S6a–d), providing direct evidence that these pathways are sensitized in experimental latency models *in vitro*.

To assess how viral protein expression contributed to increased stress kinase signaling, we analyzed anisomycin-stimulated p-p38 and p-JNK signaling simultaneously with Gag p24 expression by flow cytometry. Latently infected (p24−) T_CM_ cells (Fig. 5c) exhibited increased levels of p-p38 and p-JNK within 15 minutes of anisomycin stimulation relative to uninfected cells (Fig. 5d,e), demonstrating that latent infection confers sensitivity to p38 and JNK signaling. Levels of p-JNK were even higher in infected virus-expressing (p24+) T_CM_ cells, though these cells did not show increased levels of p-p38 compared to latent T_CM_ cells (Fig. 5d,e). Flow cytometry analysis comparing the Jurkat and J-DHIV cells (Fig. 5f) yielded similar results, with p-p38 and p-JNK signaling significantly higher in the latent GFP- fraction within 15 minutes of anisomycin stimulation (Fig. 5g,h). Interestingly, virus-expressing GFP+ J-DHIV exhibited greater increases in p-p38 and p-JNK than primary T_CM_ cells and these increases were sustained for at least 1 hour after anisomycin stimulation (Fig. 5g,h). Overall, we conclude that, latent infection sensitizes cells to p38 and JNK stress kinase signaling, and viral protein expression can increase this sensitization.

**Figure 6:**
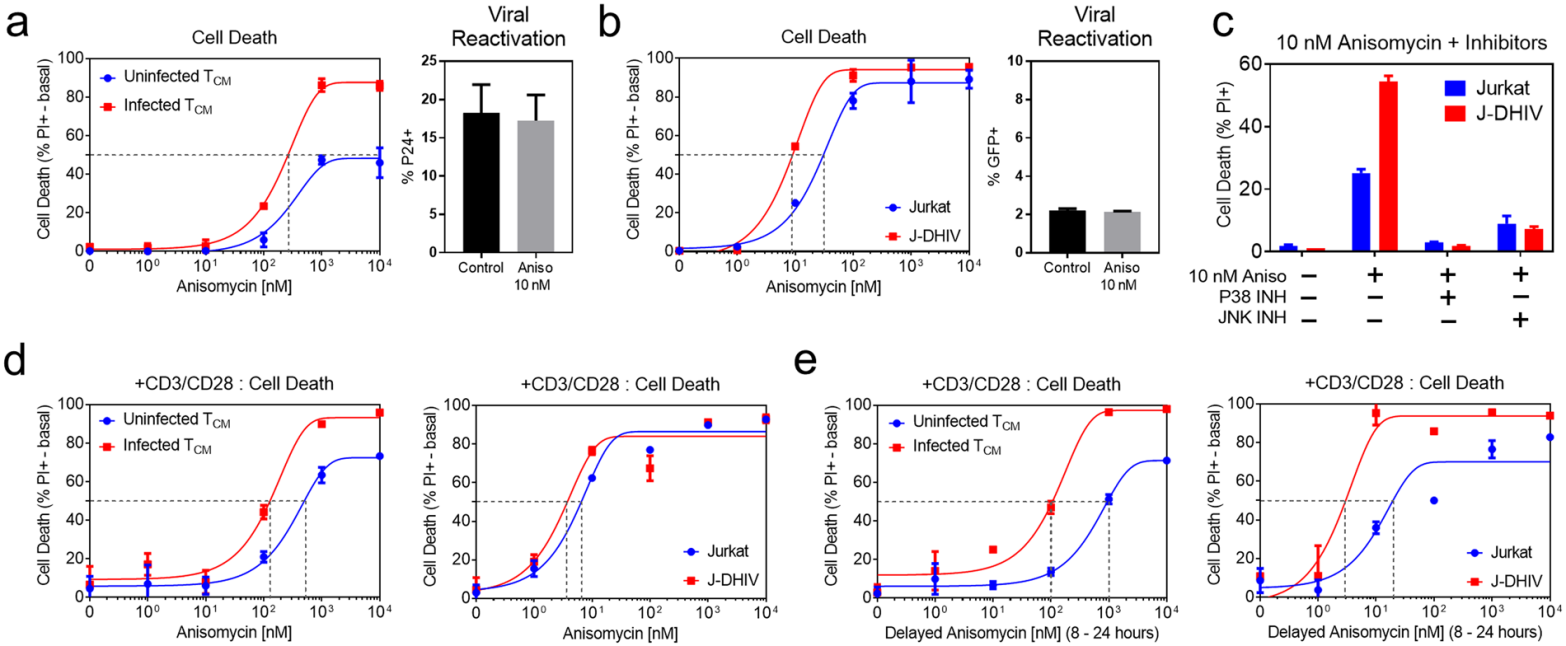
Latently infected cells exhibit increased sensitivity to cell death via the p38 and JNK agonist anisomycin. (**a**,**b**) Cell death measured at 24 hours in response to varying doses of anisomycin (left plots) in uninfected (blue) and infected (red) cells for primary T_CM_ cells (**a**) and Jurkat/J-DHIV cell lines (**b**). Basal responses were subtracted and the percentage of the viable cells killed in response to anisomycin is reported. Data presented as means ± SD and fit with a non-linear curve (see Methods). Viral reactivation in response to 10 nM anisomycin measured by flow cytometry (right plots; % p24 + in primary T_CM_ cells and % GFP+ in the J-DHIV cell line model). Percentage of cells expressing p24 or GFP at 24 hours was measured in biological duplicate. Data presented as means ± SD. (**c**) Cell death measured in uninfected Jurkat (blue) and J-DHIV (red) after 24 hours of exposure to 10 nM anisomycin +/− p38 or JNK inhibitors. (**d**) Cell death measured at 24 hours in response to CD3/CD28 stimulation and varying doses of anisomycin in uninfected (blue) and infected (red) cells for primary T_CM_ cells and Jurkat/J-DHIV cell lines. (**e**) Cell death measured at 24 hours in response to CD3/CD28 stimulation and varying doses of anisomycin spiked in at 8 hours in uninfected (blue) and infected (red) cells for primary T_CM_ cells and Jurkat/J-DHIV cell lines.

We further found that primary infected T_CM_ cells were more sensitive to cell death in response to anisomycin treatment, exhibiting a lower EC50 than uninfected T_CM_ cells and increased cell death even at the highest concentrations (Fig. 6a). Anisomycin did not stimulate viral reactivation, demonstrating that sensitivity to anisomycin-induced cell death was not linked to viral protein expression, and consistent with the finding that p38 and JNK signaling was increased in latently infected cells. We also observed increased sensitivity to anisomycin-induced cell death without viral reactivation in the J-DHIV cell line as compared to Jurkat cells (Fig. 6b).

We used p38 and JNK kinase inhibitors in combination with anisomycin to explore the relative contributions of p38 and JNK to the sensitization of cell death in infected cells. We found that both the inhibition of JNK and, separately, the inhibition of p38 reversed anisomycin-induced cell death in Jurkat and J-DHIV cells, but the absolute difference was greater for J-DHIV cells at anisomycin concentrations near EC50 (Fig. 6c). Thus our data suggest that both p-p38 and p-JNK contribute to the increase in anisomycin-induced cell death in latently infected cells.

Because viral protein expression appeared to increase p-JNK and, to a lesser extent, p-p38 levels, we considered whether anisomycin could further boost cell death in infected cells when combined with latent HIV reactivation. We simultaneously treated primary T_CM_ cells with CD3/CD28 and varying doses of anisomycin for 24 hours. Combining CD3/CD28 with anisomycin maintained the EC50 decrease observed for infected T_CM_ cells and J-DHIV (Fig. 6d) relative to uninfected cells, but the difference was not greater than that for anisomycin alone.

We then tested if priming latently infected cells with CD3/CD28 prior to anisomycin stimulation might be more effective at further lowering the EC50 for infected cells. Indeed, treatment with anisomycin 8 hours after CD3/CD28 stimulation resulted in a larger difference in EC50 between infected and uninfected primary T_CM_ cells (Fig. 6e), as well as a higher level of cell death in infected cells, even though the total exposure time to anisomycin was decreased. In the Jurkat/J-DHIV model, treatment with CD3/CD28 prior to adding anisomycin produced a similar EC50 reduction. Together, these data strongly suggest that latent and virus-expressing infected cells are more sensitive to pro-death signaling of p38 and JNK as compared to uninfected cells, and that activation of these pathways in a time-dependent manner can specifically increase cell death in infected cells.

## Discussion

HIV latency is a quiescent viral state that currently eludes direct host cell targeting. In this study, we pursued a novel strategy to identify changes in cell signaling that become apparent following T cell perturbation by TCR stimuli or phorbol esters, with the goal that these differences may enable selective targeting of the latent T cell reservoir. We provide evidence that latent and reactivating HIV infection is associated with changes in signal transduction in both primary cultured CD4+ T_CM_ cells and Jurkat cell line models of latency (Figs. 1–2), even though basal phosphorylation levels of the five pathways measured are indistinguishable (Supplementary Fig. S2). Our results are consistent with previous observations that pathogenic infection can alter signaling globally throughout the cell^30,40^. Moreover, we show that changes in network-level signaling result in increased sensitivity to the pro-death antibiotic anisomycin via increased phosphorylation of the p38 and JNK pathways (Figs. 5–6). These results further demonstrate that cell death can be driven selectively in latently infected cells independent of viral reactivation. Our findings motivate the exciting possibility that modified signaling pathways in latently infected cells will enable more selective “activate-and-kill” therapeutic strategies.

Previous studies analyzing cell state changes in response to latent HIV infection have largely compared latently infected and uninfected cells in the basal state (i.e., without any perturbation). While some studies demonstrated modest differences in gene expression^41^ and kinase activity^5^, these studies used long-established cell line models of latency, making it difficult to assess whether these differences arose due to cell line divergence. Studies utilizing primary latency models have focused primarily on characterizing transcriptional regulation. These transcriptome profiling studies have implicated modestly enriched surfaced markers like CD2 and CD32^6,42^ and differentially expressed genes related to p53^43^, but did not identify phenotypic differences or changes at the signaling level. Our analysis shows that it is possible to differentiate uninfected and latently infected cells based on network-level changes in phospho-signaling (Fig. 2). Importantly, we further showed that these network-level changes alter cell death, a clinically important outcome.

To our knowledge, our results provide the first evidence that latently infected cells are sensitized to cell death mediated by the p38 and JNK pathways. This finding addresses a major pitfall associated with reactivation by LRAs, which is that viral reactivation alone may not be sufficient to cause death in the latent reservoir^37^. This enhanced activation of p38 and JNK pathways, which is caused by an unknown mechanism, complements a recent report (also conducted in a cultured T_CM_ model) that p53 signaling may play a role in the establishment and maintenance of latency^43^, because p38 and JNK are known regulators of p53.

We compared phospho-signaling between uninfected and latently infected cells in both primary cultured T_CM_ and Jurkat cell models of latency, because we assumed that trends conserved across experimental latency models are more likely to represent the *in vivo* latent memory T cell reservoir. We chose a primary T cell latency model capable of generating relatively high levels of latently infected cells in order to detect signaling differences between uninfected and latently infected cells with the highest possible sensitivity. To complement this model, we used a Jurkat model of latency that responds to TCR stimulation, because such models have LRA responses that are more similar to patient T cells treated *ex vivo* than most other primary T cell models^27^. Future studies will be required to determine if stress kinase signaling is altered in latently infected cells *in vivo* and thus represents a novel clinical target for clearing latency. More broadly, our study demonstrates how systems-level approaches can improve the implementation of LRA strategies for the eradication of latent HIV infection.

## Materials and Methods

### Virus production

The DHIV-Nef + plasmid (containing a small out-of-frame deletion in the gp120-coding area that renders it defective in Env), DHIV-GFP plasmid (DHIV plasmid containing GFP in place of Nef) and the pLET-LAI plasmid (encoding an × 4 envelope gene) were kind gifts from Dr. Vincente Planelles. The envelope gene was originally derived from a CXCR4-dependent isolate, HIV-1-LAI, and the *env* glycoprotein was produced with the expression construct pLET-LAI^26,44,45^. To generate virus, DHIV-Nef + and DHIV-GFP were co-transfected with pLET-LAI plasmids into HEK293T cells (gift from Dr. David Schaffer) using standard calcium phosphate-mediated transfection. After 18 h, the transfection medium was replaced with 10 ml of fresh medium (IMDM + 10% FBS and L-glutamine in the absence of antibiotics, all from Gibco Life Technologies) and cultured for an additional 36 h. Supernatants were then collected and pre-cleared by centrifugation. After centrifugation, the supernatant was filtered, aliquoted, and frozen at −80 °C. To normalize infections, p24 was quantified in virus-containing supernatants by enzyme-linked immunosorbent assay (ELISA; ZeptoMetrix).

### Primary cultured central memory CD4+ T cells (TCM) activation and infection

Buffy coats of leukocytes were obtained from anonymous HIV- donors (New York Blood Center). Peripheral blood mononuclear cells (PBMCs) were harvested by density centrifugation with Ficoll-Paque (GE Healthcare). Naïve CD4^+^ T cells were isolated by MACS negative selection using the human naïve T-cell isolation kit (Miltenyi Biotec) to yield a population with the phenotype CD4^+^ CD45RA^+^ CD45RO^−^ CCR7^+^ CD62L^+^ CD27^+^ with purity levels equal or higher than 95%. Cells were then activated for 72 hours with beads coated with αCD3 and αCD28 antibodies, Dynabeads Human T-Activator CD3/CD28 for T cell Expansion and Activation, (ThermoFisher Scientific) under non-polarizing (NP) conditions, in the presence of 10 ng/ml of TGF-β1, 2 μg/ml of anti-Human IL-12 and 1 μg/ml of anti-Human IL-4 (all from Peprotech). Dynabeads were removed on day 3 and consequently cultured at a density of 1 × 10^6^ cells/ml in complete medium with 30 IU/ml of rhIL-2 (R&D Systems). Cells were infected by spinoculation on Day 5: 10^6^ cells were infected with 300 ng/mL p24 for 2 hours at 2900 rpm and 37 °C in 1 mL. After infection, cells kept in culture at 1 × 10^6^ cells/ml in complete medium with 30 IU/ml of rhIL-2 at 37 °C for 7 days prior to further experimentation, during which actively infected cells die, leaving a mixed population of latently infected or uninfected cells.

### Jurkat T cell culture and infection

Jurkat cells clone E6-1 (ATCC) were cultured in RPMI media (Gibco Life Technologies) supplemented with 10% FBS (Atlanta Biologicals) and penicillin and streptomycin (Gibco Life Technologies at a concentration of 2 × 10^5^−2 × 10^6^ cells/ml at 37 °C and 5% CO_2_. Cells were infected with a DHIV-GFP/X4 virus and GFP- cells were sorted to isolate uninfected and latently infected populations. Cells were then stimulated with TNFα (Peprotech), and GFP + cells were sorted into a 96-well plate to establish a clonal line of latently infected cells. This clonal cell line model provides a fully infected population with minimal divergence between uninfected and infected counterparts.

### Stimulation and inhibitor treatments

Cells were activated and stimulated with Dynabeads Human T-Activator CD3/CD28 for T cell Expansion and Activation at a 1:1 ratio (bead-to-cell) according to manufacturer protocol (ThermoFisher Scientific). Phorbol 12-Myristate 13-Acetate (Sigma Aldrich) was used at 10 nM. Ionomycin from Streptomyces conglabatus (Sigma Aldrich) was used at 1 μM. Prostratin (Santa Cruz Biotechnology) and Suberoylanilide Hydroxamic Acid (SAHA, Santa Cruz Biotechnology) were both were used at a concentration of 1 μM. For inhibitor experiments, cells were pre-treated with the inhibitor for one hour prior to stimulation. Inhibitors were used at the following concentration: PD 98059 (Calbiochem) at 10 μM, LY 294002 (Calbiochem) at 50 μM, SB 203580 (Calbiochem) at 10 μM, SP 600125 (Calbiochem) at 50 μM, and ACHP (Tocris Bioscience) at 5 μM. Reported values for inhibitor treatments were normalized to the maximum levels of viral activation and cell death induced by CD3/CD28 or PMA/I treatment to account for variation across infections. Dose response curves were collected after 24 hours of stimulation with anisomycin from Streptomyces griseolus (Sigma Aldrich) in 10-fold serial dilutions ranging from 1 nM to 10 μM.

### Cell signaling time course collection

Treated cells were lysed in RIPA buffer and lysates were stored at −80 °C. Lysate concentrations were normalized and diluted to 10 μg/well for signaling measurements. Phospho-protein levels were measured using bead-based xMap immunoassays (Bio-plex, from Bio-Rad) according to manufacturer protocols. Beads specific for p-ERK, p-AKT, p-p38, p-JNK, and p-IκB-α were combined and incubated with lysate samples overnight at room temperature. The following day, beads were washed, incubated with detection antibody, and streptavidin-PE prior to analysis on the Bio-Plex 200 system (Bio-Rad).

### Flow cytometry assays

Cells were fixed with and permeabilized with the BD Cytofix/Cytoperm solution (BD Biosciences). Cells were washed and stained in the presence of BD Perm/Wash Buffer (BD Biosciences). For primary cells, viral activation was measured by intracellular staining for p24, an HIV-1 capsid protein, with the monoclonal antibody AG3.0 at a 1:40 dilution (NIH AIDS Reagent Program, courtesy of Dr. Jonathan Allan) and anti-mouse IgG secondary antibodies conjugated to AlexaFluor 647 (ThermoFisher Scientific). For Jurkat latent infections, activation was quantified by analyzing green fluorescent protein (GFP) level in 10,000 cells. Cell death was measured after 10 minutes of staining with propidium iodide (Sigma Aldrich) at a working concentration of 5 μg/ml. Flow cytometry assays were analyzed on an Accuri C6 Cytometer (BD Biosciences).

### Western Blotting

Cells were treated with 1 μM anisomycin for 30 minutes. Cells were pelleted and lysed in RIPA buffer supplemented with protease and phosphatase inhibitors (Roche). BCA assays were used to normalize total protein loading per sample and proteins were separated via SDS-PAGE and transferred onto a nitrocellulose membrane. Membranes were blocked with Odyssey Blocking Buffer (Rockland Inc.) for 1 hour at room temperature and incubated with primary antibodies (Cell Signaling Technologies: p-p38 (9211) and p-JNK (9251)^46^ or Abcam: (ab89984)^47^) at 4 °C overnight. Secondary antibodies (Li-COR) were applied for 1 hour and fluorescent signal was quantified using a Li-COR Odyssey Imaging System.

### Partial least squares discriminant analysis (PLS-DA) and Partial Least Square Regression analysis

PLS-DA and PLSR were performed with SIMCA software (Umetrics). Phosphorylation data were normalized to the maximum average measurement of each protein per donor pair (uninfected and infected cells) per stimulation condition. The number of principal components was optimally calibrated to minimize root mean square error (RMSE) and maximize predictive ability. Variables that contributed least to the model (with a VIP < 1) were removed to improve the predictive ability, indicated by Q^2^. Orthogonal signal correction was used to improve PLS-DA model interpretability. PLSR model uncertainty was estimated by jack-knifing^33^.

### Statistical analyses and curve fitting

Significance for inhibitor testing was determined per experiment, then tabulated cumulatively across all infections (different donors and different days). To account for this variability, only measurements that were significantly different across a majority of repeated trials are marked as significant. Dose-response curves to the agonist anisomycin ± CD3/CD28 stimulation were fit with a non-linear regression curve (GraphPad Prism). Cell death responses in the absence of anisomycin ([anisomycin] = 0 nM) were subtracted from samples treated with anisomycin and then normalized to the percentage of remaining viable cells (i.e. 100%-%[anisomycin = 0 nM]) to determine the percentage of the remaining viable cells killed in response to anisomycin. Error bars show standard deviation of two biological replicates.

### Data Availability

The datasets generated and analyzed during this study are included in the Supplementary Information files for this publication.

### Ethics Statement

All donors used in this study were anonymous and therefore not considered to be Human Subjects Research, as determined by the Human Investigation Committee at Yale University, pursuant to the Office of Human Research Protections definition of human subject.

## Electronic supplementary material

Supplementary Information 

S1

S2

S3

S4

S5

S6

S7

S8

S9

S10

S11

S12
