# Supplementary material for: Systems analysis of latent HIV reversal reveals altered stress kinase signaling and increased cell death in infected T cells
Source: Sci Rep. 2017 Nov 23;7:16179. doi: 10.1038/s41598-017-15532-0 (PMC5701066; doi:10.1038/s41598-017-15532-0)
Supplement: Supplementary file 1 — Supplementary Information [file 41598_2017_15532_MOESM1_ESM.pdf]

Systems analysis of latent HIV reversal reveals altered stress kinase signaling and increased cell death in infected T cells

Linda E. Fong, Endah S. Sulistijo, and Kathryn Miller-Jensen*

Supplementary Information

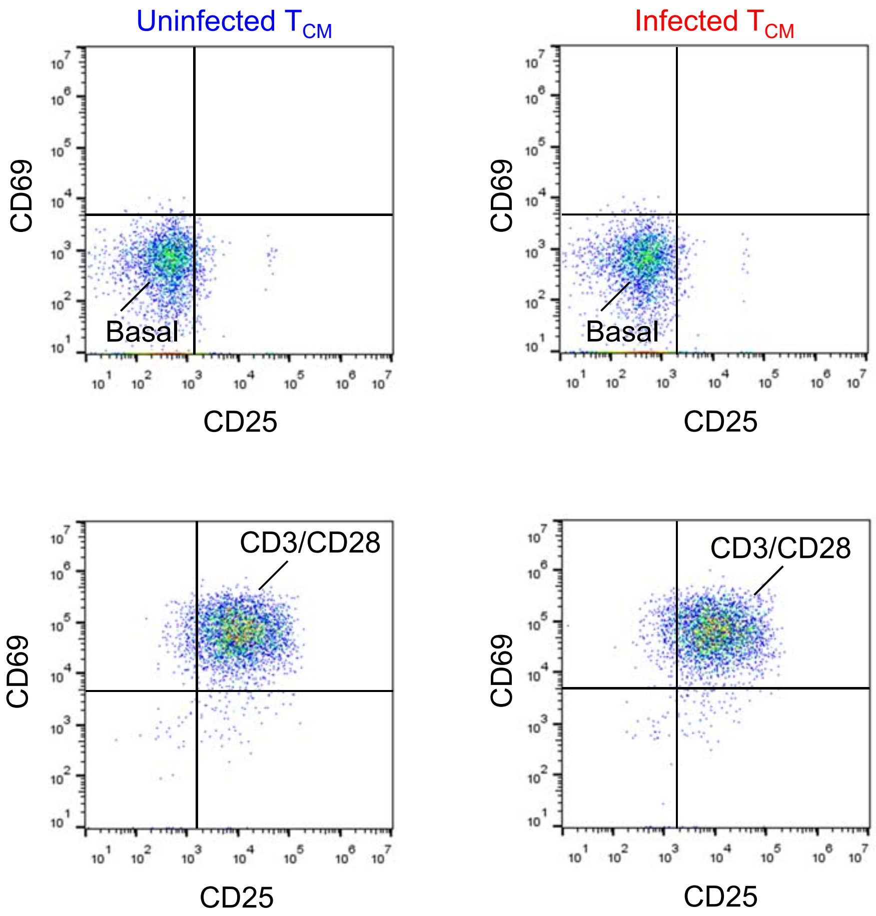

Supplementary Figure S1. CD3/CD28 stimulation induces T cell activation similarly in uninfected and latently infected cells. Uninfected and infected primary cultured CD4+ T_{CM} populations were co-stained for the activation markers, CD25 and CD69, in the basal state and following 48 hours of CD3/CD28 stimulation and measured by flow cytometry.

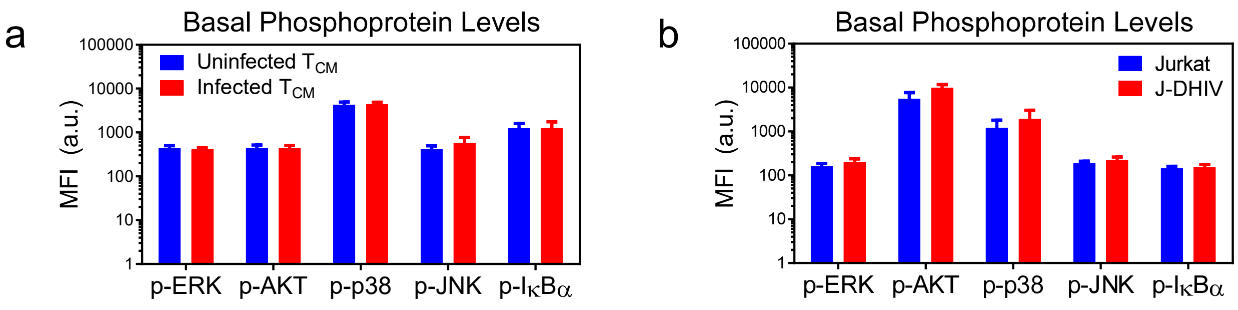

Supplementary Figure S2. Latently infected cells and uninfected cells show similar levels of basal phosphorylation. (a-b) Basal phosphorylation levels of ERK, AKT, p38, JNK, and IκBα as measured by bead-based immunoassay for uninfected (blue) and infected (red) primary T_{CM} cells from one representative donor (a) and for Jurkat (blue) and J-DHIV (red) T cells (b). Data are plotted as means ± standard deviations (SD) of at least 3 biological replicates.

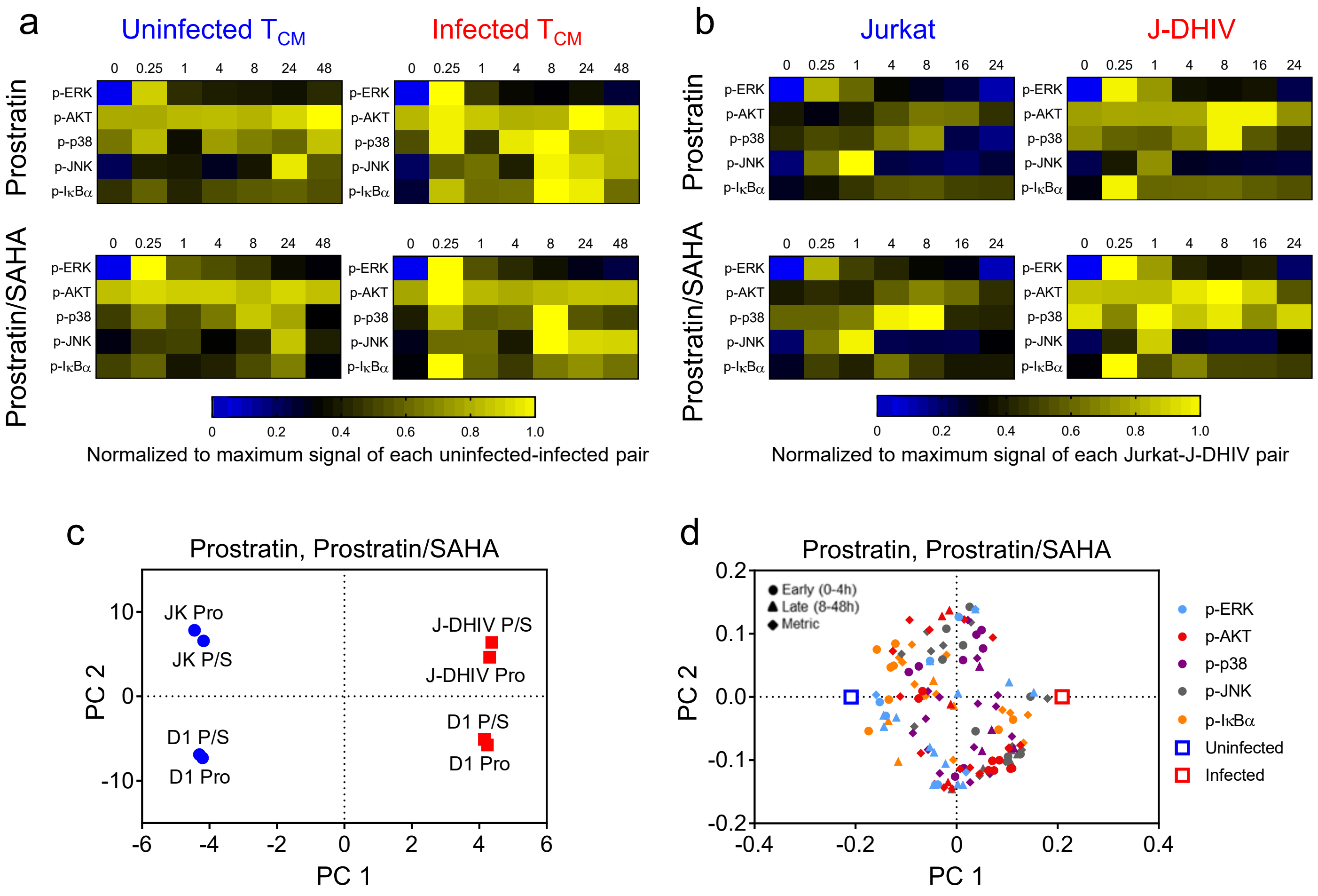

Supplementary Figure S3. Phospho-signaling differences following stimulation with prostratin +/- SAHA classify uninfected versus latent HIV-infected T cells in a PLS-DA model. (a-b) Heatmaps of phospho-protein time courses measured by bead-based immunoassay following Prostratin or Prostratin/SAHA stimulation in uninfected (blue) and infected cells (red) for (a) primary T_{CM} cells from one donor and (b) Jurkat and J-DHIV cell lines. Phospho-signals are normalized to the maximum level of each uninfected and infected pair. Phospho-signals were measured in biological triplicate and 90% of measurements had an SD < 15%. Raw data provided in **Supplementary Datasets S9-S12**. (c-d) PLS-DA model comprising three principal components was calculated from phospho-protein time course data presented in (a-b) for uninfected and infected T cells treated with Prostratin and Pro/SAHA (R2X = 0.71, R2Y = 0.99, Q2 = 0.82). (c) Model scores of uninfected (blue) and infected (red) cells for both primary T_{CM} cells and Jurkat/J-DHIV cell lines projected onto PC1 versus PC2. (d) Model loadings of phospho-proteins and host-cell classification (uninfected, blue; infected, red) projected onto PC1 versus PC2 for each phospho-protein (see legend for color codes). Early time points (0-4 hours), circles; late time points (8-48 hours), triangles; time-dependent metrics, squares.

a

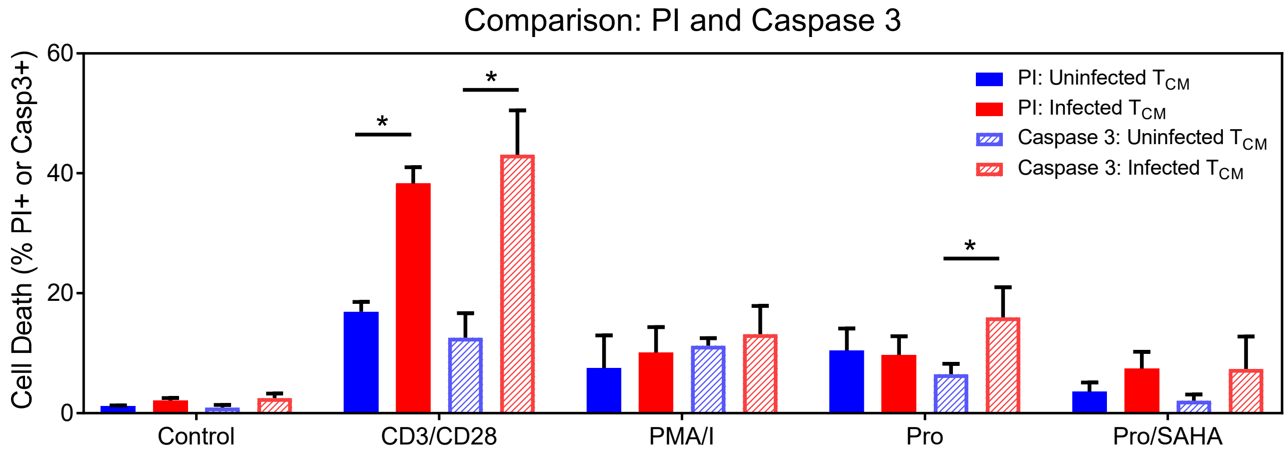

Supplementary Figure S4. PI measurements correlate closely to apoptosis levels as measured by cleaved caspase 3 immunostaining. (a) Uninfected and infected primary T_{CM} cells were treated with the indicated stimuli and cell death was assayed via PI staining (same as data presented in **Figure 4a**) or cleaved caspase 3 immunostaining and measured by flow cytometry. Percentage of positively stained cells at 24 hours was measured in biological duplicate. Bar graph depicts means ± SD.

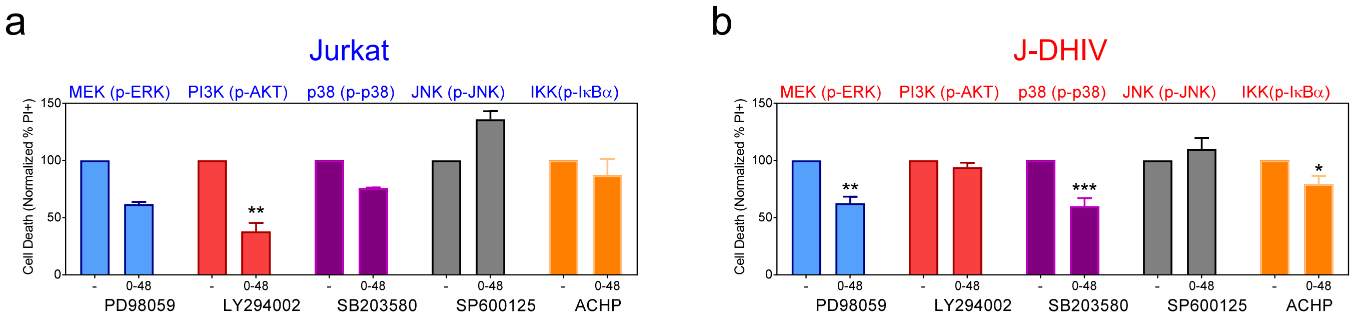

Supplementary Figure S5. Pathway inhibition in Jurkat/DHIV cell lines emphasizes the role of p38 for cell death regulation. Cell death in the presence of the indicated inhibitor was measured in response to stimulation with CD3/CD28 in Jurkat (a) and J-DHIV cells (b) by flow cytometry and normalized to the uninhibited control. Percentage of PI+ cells was measured in biological triplicate. Data are presented as means ± SD. Student t-tests were conducted to determine significance (***, p ≤ 0.001; **, p ≤ 0.01, * p ≤ 0.05, ns, p >0.05).

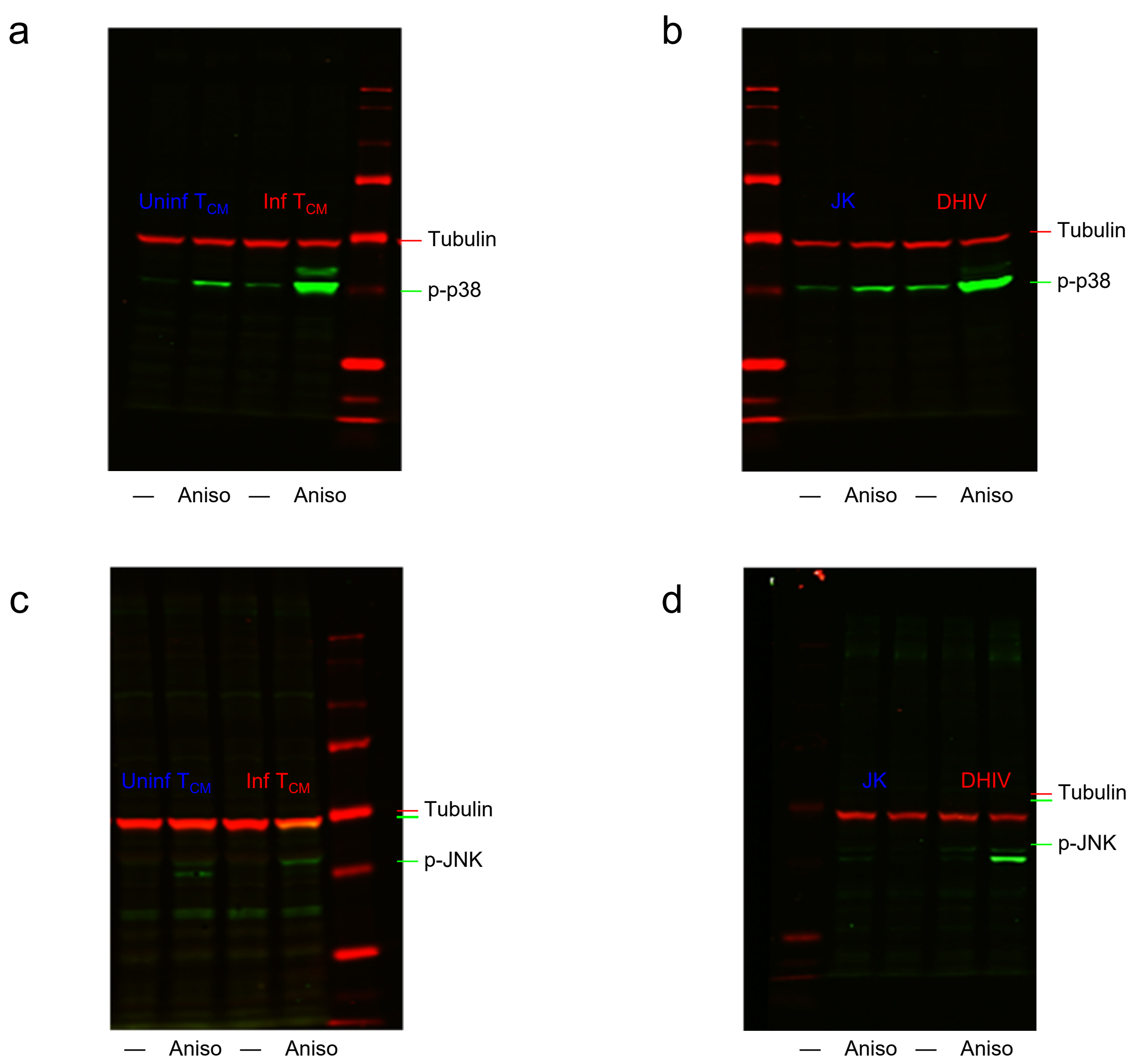

Supplementary Figure S6. Western blots show that latently infected cells exhibit increased signaling following treatment with the p38 and JNK agonist anisomycin. (a-d) Western blots for p-p38 (a-b) and p-JNK (c-d) expression with and without anisomycin treatment in uninfected (blue, left 2 lanes) and infected (red, right 2 lanes) primary T_{CM} cells (a,c) and Jurkat (JK) /J-DHIV cells (b,d).
Antibodies from Cell Signaling Technologies: Anti- p-p38 (Cat # 9211S) [46] *and Anti- p-JNK (Cat # 9251S)* [46]
Antibody from Abcam: Anti- alpha Tubulin Loading Control (Cat # ab89984) [47]

Phospho-protein	Residue Measured	Relevance to HIV
p-ERK	Thr^{202}/Tyr^{204}<br>Thr^{185}/Tyr^{187}	• ERK phosphorylation of the transcription factor LSF reduces binding at the HIV LTR, thereby decreasing recruitment of YY1 and histone deacetylases, to promote HIV reactivation (Ylisastigui, L. J Virol. 2005).<br>• ERK phosphorylates transcription factors c-Myc, AP-1, NF-κB, IL-6, ATF-2, and ELK-1 which contribute to HIV expression (Yang, X. J Bio Chem. 1999).<br>• ERK inhibitor U0126 blocks both P-TEFb recruitment to the HIV LTR, reducing HIV transcription (Briant, L. J Immuno. 1998).
p-AKT	Ser^{473}	• SAHA induced acetylation and NF-κB activation is AKT-dependent. Treatment of primary cells with PI3K inhibitor Wortmannin has shown no effect on HIV reactivation (Contreras, X. PLoS Pathogens. 2009).<br>• Nef-mediated HIV reactivation is associated with AKT activation. Treatment with protease inhibitors, but not RTIs, can block this AKT activation. (Kumar,A et al. Sci Rep. 2016)
p-p38	Thr^{180}/Thr^{182}	• p38 phosphorylation increases LSF occupancy at LTR, promoting HIV transcription and p38 inhibition blocks the ability of YY1 to inhibit HIV activation (Ylisastigui 2005).
p-JNK	Thr^{183}/Tyr^{185}	• Treatment of primary cells with JNK Inhibitor V, AS601245, prevents HIV reactivation (Wolschendorf, F. J Virol. 2012).
p-IκBα	Ser^{32}/Ser^{36}	• IKK-dependent phosphorylation and degradation of IκBα leads to subsequent release and nuclear translocation of the NF-κB transcription factor, which positively regulates HIV transcription (Brooks, D. PNAS. 2003).<br>• In CCL19-resting CD4+ T cells, inhibition of AP-1 and NF-κB inhibits HIV integration. (Saleh, S et al. Retrovirology. 2016)

Supplementary Table S1. Phosphorylation sites and phospho-protein involvement in HIV activation and expression.

Estimated Latent Percentage	Donor 1	Donor 2	Donor 3
Fig. 1e	17.3 ± 5.8	42.9 ± 1.3	48.1 ± 1.8
Fig. 1f	36.1 ± 3.7	36.9 ± 2.9	45.2 ± 2.1
Fig. 3a, c	17.3 ± 5.8	42.9 ± 1.3	48.1 ± 1.8
Fig. 3b, d	36.1 ± 3.7	36.9 ± 2.9	45.2 ± 2.1
Fig. 4a-b	17.3 ± 5.8	42.9 ± 1.3	48.1 ± 1.8
Fig. 4c-d	36.1 ± 3.7	36.9 ± 2.9	45.2 ± 2.1
Fig. 4e-f	22.6 ± 1.3<br>34.4 ± 4.5<br>43.7 ± 3.2	32.7 ± 3.3<br>55.6 ± 3.7<br>47.0 ± 4.8	
Fig. 5a		39.2 ± 1.7	
Fig. 5a-d	17.3 ± 5.8	42.9 ± 1.3	48.1 ± 1.8
Fig. 5e-f	22.6 ± 1.3<br>34.4 ± 4.5<br>43.7 ± 3.2	32.7 ± 3.3<br>55.6 ± 3.7<br>47.0 ± 4.8	
Fig. 6c, g, k		54.2 ±6.9	
Fig. 6f, j		36.2 ± 2.6	
S2 Fig. a	17.3 ± 5.8<br>36.1 ± 3.7	42.9 ± 1.3<br>36.9 ± 2.9	48.1 ± 1.8<br>45.2 ± 2.1
S3 Fig. a-d	25.97 ± 0.7		
S4 Fig. a	25.97 ± 0.7		
S4 Fig. b	17.3 ± 5.8<br>36.1 ± 3.7<br>25.97 ± 0.7		
S6 Fig. a-f		54.2 ± 6.9	
S7 Fig. a-b		36.2 ± 2.6	
S8 Fig. a-b	36.1 ± 3.7	36.9 ± 2.9	45.2 ± 2.1
S8 Fig. c-d	25.97 ± 0.7		

Supplementary Table S2. Estimated percentage of latent cells per donor per experiment based on the response to CD3/CD28 stimulation.
